# A pilot study of the mistletoe and breast cancer (MAB) trial: a protocol for a randomised double-blind controlled trial

**DOI:** 10.1186/s40814-022-01036-w

**Published:** 2022-04-06

**Authors:** Susan Bryant, Lorna Duncan, Gene Feder, Alyson L. Huntley

**Affiliations:** Centre for Academic Primary Care, University of Bristol, Canynge Hall, Bristol, BS8 2PS UK

**Keywords:** Mistletoe therapy, Herbal, Breast cancer, RCT, Quality of life, Fatigue

## Abstract

**Background:**

A Cochrane review of mistletoe therapy concludes that there is some evidence that mistletoe extracts may offer benefits on measures of quality of life during chemotherapy for breast cancer, but these results need replication. Our aim is to add to this evidence base by initially testing the feasibility of a UK pilot placebo-controlled, double-blind randomised controlled trial of mistletoe therapy in patients with breast cancer undergoing chemotherapy with or without radiotherapy.

**Methods/design:**

A mixed phase pilot placebo-controlled, double-blind randomised controlled trial of mistletoe therapy in patients with breast cancer (EudraCT number: 2018-000279-34). There will be three arms (groups) in the trial: Iscador M, Iscador P, with physiological saline as the placebo. The aim is to recruit 45 adult patients with a new diagnosis of early or locally advanced breast cancer, up to 12 weeks following definitive breast surgery whose standard treatment plan includes chemotherapy with or without radiotherapy. They will be taught to administer the mistletoe and breast cancer (MAB) therapies subcutaneously. MAB therapy will continue throughout their standard chemotherapy and radiotherapy and 1 month beyond. The main outcome of the MAB study is the feasibility of conducting such a trial within the NHS in order to inform a future fully powered investigative trial. Feasibility will be measured through recruitment, retention and patient experience using clinical research forms, patient diaries, cancer-related questionnaires and qualitative interviews conducted with both patients and oncology staff.

**Discussion:**

This trial is the first of its kind in the UK. Currently, mistletoe therapy is mostly available through private practice in the UK. Completion of this feasibility study will support applications for further funding for a fully powered randomised controlled trial which will measure effectiveness and cost-effectiveness of this herbal therapy.

## Background

In Europe, *Viscum album* L. (mistletoe) is the most commonly used therapy by patients with cancer and is integrated into conventional oncology treatment programmes in Germany, Switzerland and Holland. Despite this use of mistletoe therapy, it is only relatively recently that it has been the subject of randomised controlled trials (RCTs), although few are placebo-controlled trials. A Cochrane review conducted in 2008 concluded that the effects of mistletoe on the adverse effects of chemotherapy and radiotherapy were a reduction of these effects and/or improvement of quality of life (QOL) in breast cancer patients, but that the magnitude of these effects could not be reliably estimated from current trials and was not pooled in a meta-analysis [1]. A 2019 systematic review included 28 publications of mistletoe therapy for cancer which included 17 RCTs that report quality-of-life outcomes [2]. The authors conclude ‘to date, no clear statement regarding the efficacy of mistletoe treatment can be derived from randomised controlled studies’. A 2020 systematic review of mistletoe therapy for cancer patients included 26 publications with 30 data sets from RCTs and controlled trials in a meta-analysis [3]. They acknowledge the heterogeneity of the data and report a pooled standardised mean difference for global quality of life (all cancers) after treatment with mistletoe extracts vs. control (*d* = 0.61 (95% *CI* 0.41–0.8181, *p* < 0.00001)). Both reviews acknowledge the limitations of the data and the uncertainties surrounding the lack of placebo-controlled trials in mistletoe therapy. This uncertainty is coupled with the fact that potential recruitment of cancer patients into placebo controlled RCTs of mistletoe on mainland Europe is limited by the popularity of the therapy. This is not the case in the UK, where trial participants are likely to be naïve to mistletoe as a therapeutic agent. Whilst mistletoe can be prescribed in the UK, this is mostly through private practice, despite its potential to improve the patient experience of cancer care, a major priority of the NHS cancer plan [4]. The primary aim of this external mixed phase pilot trial, the first of its kind in the UK, is to test the feasibility of a placebo-controlled, double-blind RCT of mistletoe therapy in patients with breast cancer undergoing chemotherapy with or without radiotherapy in the NHS setting.

## Methods

### Design

The design is a pilot mixed phase, placebo controlled, double blind external RCT of mistletoe therapy in patients with breast cancer undergoing chemotherapy with or without radiotherapy. The primary aim of feasibility of the trial will be considered in terms of recruitment, retention, attrition, blinding and acceptability to patients and health professionals. There will be three arms (groups) in the trial: Iscador M, Iscador P (mistletoe therapy) with physiological saline as the placebo.

This mixed phase pilot study is an initial step to explore the innovative provision of mistletoe therapy within conventional NHS cancer care. Our primary aim is to inform feasibility and identify modifications needed in the design of a larger trial, ensuring hypothesis testing.

### Participants

We aim to recruit 45 adult patients with a new diagnosis of early or locally advanced breast cancer, up to 12 weeks following definitive breast surgery whose standard treatment plan includes chemotherapy with or without radiotherapy. The patients in this feasibility trial will be recruited via one site the Bristol Haematology and Oncology Centre (BHOC) at University Hospitals Bristol NHS Foundation Trust. 

### Inclusion and exclusion criteria

Potential participants will include adults 18 years or over with histologically verified early or locally advanced invasive breast cancer and with planned adjuvant chemotherapy, with or without radiotherapy and able to be randomised within 12 weeks of surgery. Patients who are to receive only radiotherapy will be excluded as this treatment is generally well tolerated and of short duration. They must be willing to self-administer or have a nominated person administer injections. Their Eastern Cooperative Oncology Group (ECOG) performance status must be 0 or 1, and they should have no active, uncontrolled infection. Female participants of childbearing age must be willing to adopt adequate contraceptive measures, and males must follow the chemotherapy guidance of the BHOC with regard to contraception. Patients will be excluded if they are receiving immunomodulatory therapy, receiving endocrine therapy as a stand-alone treatment, have previously had invasive breast cancer or bilateral breast cancer or have chronic viral infections such as hepatitis B and C and HIV known allergy to mistletoe, or be using/have had mistletoe within the last 5 years, acute inflammatory or pyrexial conditions, chronic granulomatous disease, active auto-immune diseases, or hyperthyroidism with tachycardia. Where appropriate, patients who are recommended to receive trastuzumab and/or endocrine therapy, as well as chemotherapy, are eligible.

### Trial registration and ethical approval

EudraCT number: 2018-000279-34

All necessary research governance approvals were sought and approved including REC reference: 18/SW/0045/date of favourable opinion 12/04/2018.

### Randomisation and blinding (Fig. 1)

It was decided to proceed with a three-arm trial for two reasons: (a) both Iscador® M and Iscador® P are recommended by Iscador AG (https://www.iscador.com/de/) for treatment of breast cancer with no evidence for either one being superior to the other, and (b) by having a 1:1:1 randomisation regime, the participants involved will get a better chance of receiving mistletoe therapy, and therefore, it may enhance recruitment.

**Fig. 1:**
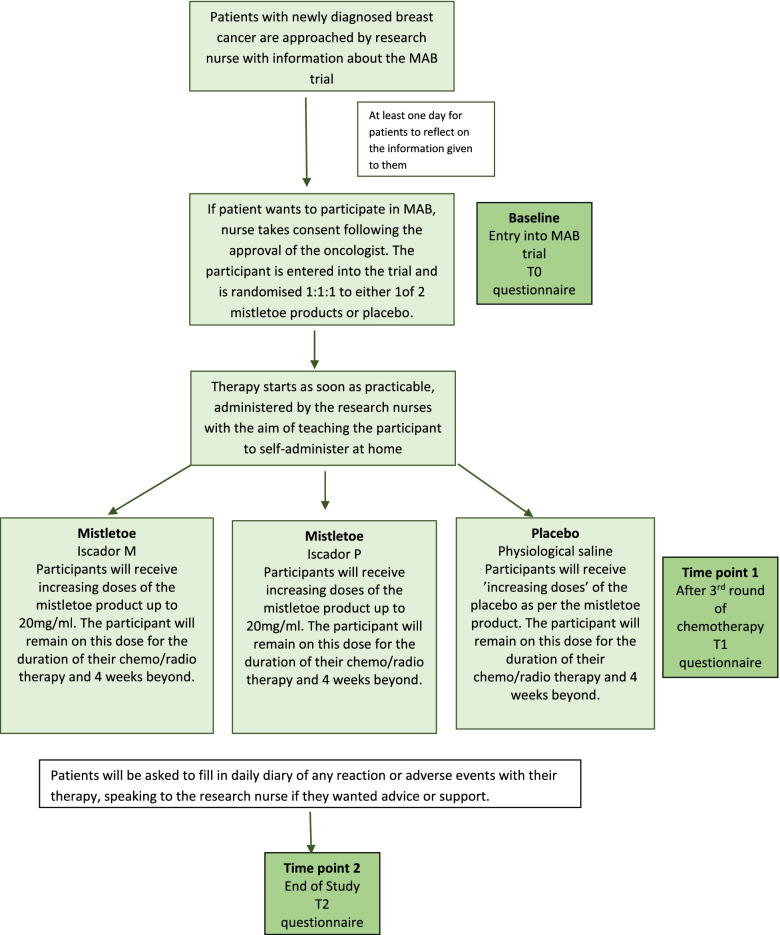
Flow chart of MAB protocol

The patient randomisation list and the medication block randomisation lists will be produced by an in-house statistician at Iscador AG. Randomisation of patients will be conducted by the University Hospitals Bristol Pharmacy (UHBP). Allocation of participants to Iscador® M/Iscador® P/control 1:1:1 ratio will be performed by the UHBP. A separate randomisation list will be held by UHBP for individual emergency unblinding. In the case of a serious adverse event and unblinding being required, the pharmacist will be asked by the principal investigator to look at the unblinding randomisation list using the package coding of the prescription without resulting in the unblinding of all the other patients due to the block randomisation. Both participants and healthcare professionals will be blinded to the group assignment. Any unblinding will be logged by the pharmacy. Breaking the blinding (for a single patient) will only be considered when knowledge of the treatment assignment is deemed essential by the investigator for the patient’s care.

### Intervention group

Participants will receive mistletoe preparations Iscador® M (Maleus) or Iscador® P (Pinus), and these will be available as 1 ml ampoules for subcutaneous injection. The quantity of fermented, aqueous extract from *Viscum album* L. from apple and pine tree respectively used to produce one ampoule of Iscador® product is expressed in milligrams (mgs), e.g. for one ampoule (1.0 ml of solution) of Iscador® M, 1 mg contains the extract of 1 mg fermented apple tree mistletoe. The proposed dose escalating regime is outlined in Table 1. This standard therapy regime was devised from the manufacturer’s recommendation in conjunction with the MAB advisory group. A participant’s therapy is increased from the lowest dose to their optimal dose. The optimal dose for a participant is a dose at which they experience a sustained local skin reaction, still present 24 h after the injection. Such a reaction determines this dose as the dose they remain on for the rest of their trial treatment unless they develop sustained skin reaction of > 5 cm. In cases of skin reactions of > 5 cm, the participants reduce their dose as indicated in Table 1, and this becomes their optimal dose. If a participant does not have a reaction at the maximum dose of 20 mg/ml, that becomes their optimal dose.

**Table 1:** Example of typical study therapy and maintenance regime for both Iscador® M (Maleus) and Iscador® P (Pinus)

Induction phase	
**Week 1**	0.01 mg (1.0 ml) × 3 = total of 0.03 mg Iscador M or P
**Week 2**	0.1 mg (1.0 ml) × 3 = total of 0.3 mg of Iscador M or P
**Week 3**	1 mg (1.0 ml) × 3 = total of 3 mg of Iscador M or P
**Week 4**	10 mg (1.0 ml) × 3 = total of 30 mg of Iscador M or P
**Week 5**	20 mg (1.0 ml) × 3 = total of 60 mg of Iscador M or P

### Placebo (control group)

The 1 ml placebo ampoules will have identical external packaging and labelling as the mistletoe ampoules. The placebo is physiological saline 0.90% w/v of sodium chloride, 308 mOsm/L or 9.0 g per litre. Physiological saline has been prepared by the manufacturer of the study medication according to good manufacturing process criteria [5].

### Treatment regime

The study medication will be increased from the lowest dose by the research nurse using the standardised regime until an optimal dose is achieved, and then the participant will stay on this dose for the rest of the study (Table 1). An identical procedure will be carried out with both the Iscador and the saline products. However if the participant is randomised to saline, the same dose will be administered throughout (physiological saline 0.90% w/v of sodium chloride, (1.0 ml)). There is unlikely to be a sustained local reaction with the saline placebo, but essentially, the same rule applies as for the mistletoe arm: the participant would continue on the same saline preparation in week five.

### Outcome measures

Feasibility will be measured using mixed methods to assess the following outcomes: recruitment, adherence, acceptability, adverse events, completion of patient outcomes, attrition, blinding and therapy-related symptoms. These are detailed in Table 2 along with the mode of measurement, type of data, measurement of success and analysis plan.

**Table 2:** Assessment of the primary aim of the feasibility of the MAB study

Feasibility outcome	Mode of measurement	Quantitative/qualitative	Measurement of success	Analysis
**Recruitment rate**	Clinical report form	Quantitative	Meeting our recruitment target of 45 participants	Narrative numerical reporting
**Specific obstacles to recruitment**	a) Recruitment log b) Qualitative interviews with health professionals	Quantitative and qualitative	a) Complete record of recruitment with reasons for not meeting inclusion criteria or individuals’ reasons for refusal b) Identification of the obstacles to recruitment from health professionals	a) Narrative numerical reporting of recruitment with reasons b) Narrative description of the overarching obstacles identified by health professionals
**Adherence to the study therapy schedule**	a) Clinical report form b) Participants’ diaries	Quantitative	a) Completion of clinical report form b) Completion of diaries and completion of sections with diaries	a) Narrative numerical reporting of completed CRF as participants progressed through trial b) Percentage completion of diaries and completion of items within individual diary sheets (3 per week)
**Acceptability of regular subcutaneous injections**	a) Patient diaries b) Qualitative study	Qualitative	Identification of factors associated with acceptability or not of injection regimes	a) Narrative thematic and numerical report of acceptability b) Formal qualitative analysis from participants and health professional qualitative interviews
**Adverse events from mistletoe and placebo subcutaneous injections**	a) Clinical report form b) (Serious) adverse events report forms	Quantitative	No serious adverse events reported No unexpected or unusual adverse events with mistletoe therapy	a) Narrative numerical reporting b) Numerical and descriptive categories of adverse events and serious adverse events
**Completion of outcome measures**	Participants’ questionnaires	Quantitative	Completion of patient questionnaire and completion of questions within questionnaire	Narrative numerical reporting (%) completion of questionnaires plus % of questions completed within them
**Attrition rate with reasons if possible**	Clinical report form/withdrawal form	Quantitative and qualitative	Reporting of withdrawals and dropouts with reasons	Narrative numerical reporting of withdrawals/dropouts with reasons
**Assessment of blinding of patients**	a) Questions in final questionnaire b) Qualitative interviews and possibly diaries	Quantitative and qualitative	Definitive opinion of the participants with reasons	a) Narrative numerical reporting by participants of whether they thought they were receiving mistletoe/placebo or did not know plus reasons b) Narrative reporting of additional reasons for identifying allocation of treatment
**Assessment of therapy-related symptoms and health-related quality of life in the sample population**	a) Participants’ diaries b) Qualitative interviews	Qualitative	Identification of therapy-related symptoms and general quality-of-life descriptions	a) Narrative thematic and numerical report of symptoms and descriptions of quality of life b) Formal qualitative analysis from participants

### Participant diaries and questionnaires

Participants will receive a diary card pack to record their study therapy and responses three times a week to correspond with the MAB study regime.

The MAB questionnaire pack comprises six questionnaires and will be administered at three time points during the trial: *time point 0 or baseline* — following randomisation and before the start of chemotherapy regime; *time point one* — following the 3rd cycle of chemotherapy; and *time point two —* 4 weeks after the last standard treatment (chemotherapy with or without radiotherapy), on the day of the last study treatment. The questionnaires included are as follows:

1. European Organization for Research and Treatment of Cancer (EORTC) QLQ-C30 (quality of life — cancer 30 items) questionnaire
2. EORTC QLQ-BR23 (quality of life — breast cancer 23 items) questionnaire [6, 7]
3. Functional Assessment of Cancer Therapy-Neutropenia (FACT-N) scale [8]
4. Cancer fatigue scale [9]
5. Autonomic regulation scale [10]
6. The CompleMentary and Alternative Beliefs Inventory (CAMBI) [11]

### Participant and staff interviews

Semi-structured interviews will be conducted with MAB study participants and BHOC staff to explore acceptability of the MAB therapy, therapy-related symptoms and administration of/participation in the trial. Interviewees will be selected via purposive sampling where enough exist; otherwise, all participants who have indicated their consent to interview will be approached for interview, as well as relevant staff. These data will help plan the delivery and processes of the study therapy for the full trial and establish appropriate training needs. Pro-formas will be utilised in interviews, to include the following topics (as appropriate to participants and staff):

***Interview 1*** (to be completed as soon as possible after recruitment to the study in the case of participants and throughout the trial in the case of staff):

- Understanding and expectations of MAB therapy
- Awareness, interest in and use of complementary and/ or alternative therapies
- Study processes including recruitment, administration of the MAB therapy and administration and completion of diaries and questionnaires
- Local availability and perspectives on the role of CAM in cancer treatments in the NHS (staff only)

***Interview 2*** (to be completed with participants as soon as possible on completion of study participation and, if possible, towards/at the end of the trial in the case of staff):

- Further exploration of the topics in interview 1 to identify any changes/clarification and overall views on the trial.
- Participants’ understanding of the placebo effect will be investigated and their ideas on the MAB therapy treatment which they think they may have received.

### Procedures

Participation in the study will be approximately 10 months. Patient informed consent will be taken in BHOC either by the consultant or delegated by the consultant to an appropriately trained and qualified member of the research team. The patients will be given at least 24 h to consider whether they want to participate or not. If a patient decides to take part, they will be randomised into either one of the two mistletoe therapies or a placebo therapy. The first study therapy will be given within a week of randomisation to the study and, ideally, prior to the start of chemotherapy.

The participants treatment regime will be three subcutaneous injections per week. It is advised that the injections will be given every other day, followed by a 2-day break. There will be no breaks in the study therapy regime unless the participants request one, or a clinician advises one. In the case of a participant stopping their standard treatment, they will be encouraged to continue their MAB therapy during this period, but the dose will not be escalated.

Injections will be initiated by research nurses in the clinic with the aim of teaching the participant to self-administer or a nominated person (e.g. relative) to administer to the participant and continue the study therapy at home. The injections will be administered in the abdomen or thigh.

The participants will be given a booklet which contains information on self-administration of the study treatments, the expected responses and potential undesired responses and contraindications of the study therapy and a diary to record their study therapy and responses. The strength of reaction at 24 h after the injection is the indicator for either increasing or maintaining the dose. We estimate that most participants will be able to self-administer their optimal dose within 1 month (~12 visits), and some of these visits will coincide with other treatment or appointments.

### Data monitoring

The Trial Steering Committee (TSC) will also incorporate the Data Monitoring and Ethics Committee (DMEC). This group has established terms of reference and will incorporate members who are independent of the sponsor and have no competing interest.

### Sample size calculation and power

Studies suggest there is no formal way of determining numbers for a feasibility trial [12, 13]. The aim of recruiting 45 patients (15 per group) was based on both statistical and practical reasoning. The guidance by Julious is acknowledged which states that for such pilot studies, the recommendation is a sample size of 12 per group [14]. The justifications for this sample size are based on rationale about feasibility, precision about the mean and variance, and regulatory considerations. This decision was discussed with the MAB study team, its steering and advisory groups and the BHOC clinical staff. Overall, this number was chosen to allow fair assessment of the aims of recruitment, retention and completion of outcomes and an assessment of the viability of blinding allowing for some dropouts/withdrawals.

### Analysis plan

The recruitment rate will be expressed using descriptive statistics. Retention will be summarised by recording the number of participants in each study group at the pre-specified worst toxicity time point (after 3rd chemotherapy cycle, T1) and 4 weeks post standard treatment (at end of study treatment, T2). The patient-related outcome data will be summarised per each individual questionnaire (T0, T1 and T2). Completion will be noted if the form can be used. For example, the EORTC QLQ-C30 can be used if at least half the questions from the factors of interest are complete. We aim to use quality of life as our primary outcome in the planned full-scale trial and will use the suite of questionnaires in this pilot trial to determine whether we use all or some of these measures in the full trial by looking at both adherence, and participant comments within diaries, and in the qualitative interviews. We may be able to use the pilot data to determine our sample size for a full trial, but we are aware our recruitment may be too modest to allow this. Blinding will be assessed by asking the patient in their final questionnaire at T2. This will be analysed using Bang’s blinding index. Blinding will potentially be discussed in their qualitative interview if appropriate.

For the qualitative work, data from the interviews, as well as qualitative data from participant diaries and questionnaires, will be analysed thematically.

Interviews will be transcribed and coded in NVivo using themes broadly linked to those used in the interview pro-formas (recruitment; understanding/expectations of MAB therapy; views and use of complementary and/or alternative therapies, and the availability/role of these in the NHS/privately; trial recruitment/retention; MAB therapy administration/completion of diaries and questionnaires; understanding of the placebo effect; blinding; and possible improvements to the trial). Initial coding and development of themes will be performed by two members of the MAB study team (LD and AH), with remaining coding and synthesis performed by LD. Narrative synthesis of the themes from the perspectives of participants and staff will be reported using the interview data as well as relevant items from participant diaries and questionnaires.

## Discussion

Mistletoe therapy provision through the NHS is minimal with most provision occurring through private practice in the UK. This limits patient’s awareness of it and access to it. This pilot mixed phase, external RCT is the first of its kind in the UK, and completion of this feasibility study will increase awareness of mistletoe therapy for oncology patients in the UK and will support applications for further funding for a fully powered RCT which will measure effectiveness and cost-effectiveness.

The MAB study takes a mixed methods approach, not only assessing the feasibility of the trial design in a UK context but also qualitatively exploring the experience of both health professional and patient participants. The qualitative interviews will inform the design of the main trial but also expand the relatively small literature on the experience of patients receiving mistletoe therapy.

### Trial status

The current protocol is version 7.1 dated 01/05/2019; participant recruitment began on 01/08/2019 and was due to end on 31/03/2020 but was curtailed at 19/03/20 due to COVID pandemic.

## Data Availability

Not applicable — paper is a protocol.

## Abbreviations

BHOC: Bristol Haematology and Oncology Centre, UH Bristol
CRF: Case report form
RCT: Randomised controlled trial
